# A Novel Method to Characterize the Damping Capacity of EPDM/CIIR Blends Using Vibrating Rubber Balls

**DOI:** 10.3390/polym16111447

**Published:** 2024-05-21

**Authors:** Zhixin Liu, Kai Wang, Yongqiang Wu, Hanxiao Zhang, Tianyi Hao, Hongyang Qi, Bosong Liu

**Affiliations:** China Automotive Technology and Research Center Co., Ltd., Tianjin 300300, Chinaliubosong@catarc.ac.cn (B.L.)

**Keywords:** damping capacity, energy dissipation rate, EPDM/CIIR blend, characterization method

## Abstract

An experimental device fixed with a laser displacement sensor was assembled to investigate the rebound behaviors and damping mechanism of rubber balls prepared with ethylene-propylene-diene monomer (EPDM)/chlorinated butyl rubber (CIIR) blends. The result showed that a prediction model was proposed to characterize the damping capacity by using the rebound height of the rubber balls. The lower rebound height corresponded to better damping capacity. A modified equation relating to the rebound height has been obtained from the theoretical derivation on the basis of the dynamic mechanical analysis, showing that the rebound height was affected by the deformation frequency, the external excitation, and the nature of rubber blends. Furthermore, the energy dissipation rate (EDR), defined by the ratio of the height loss to the rebound time, was proposed to further characterize the damping capacity. The EDR value was shown to be highest for the pure CIIR and lowest for the pure EPDM, exhibiting a decreasing trend with the increase in EPDM content in the rubber blends. It can be expected that the damping capacity of the EPDM/CIIR blends decreases with the decrease in external excitation, the conclusion of which plays a key role in the formulation design of viscoelastic damping rubber materials.

## 1. Introduction

Rubber materials play an essential role in modern technologies, and damping is one of the most critical applications [1]. Rubber materials with hyper-elasticity and viscoelasticity show the phenomenon of the induced strain lagging behind stress when they are subjected to dynamic external force [2,3]. Experimental testing methods are significant in the research of damping materials; for example, Tang and others [4,5,6] studied the damping properties of the chlorinated butyl rubber/two-step modified montmorillonite nanocomposites and obtained an excellent damping rubber material by mechanical blending. The following are the relevant theories and testing methods proposed in the industry.

The damping performance of rubber materials is an important characterization index, and the Kelvin Vogt, Maxwell, and Zener linear viscoelastic models can characterize the damping performance of materials with very few parameters. However, these models are only theoretical models proposed under ideal conditions and cannot effectively reflect the mechanism of energy dissipation.

The Martins model [7] was proposed to evaluate the fitting accuracy of the stress–strain relationship between experimental and theoretical data for the soft tissues and nonlinear silicone rubber. The material constants for the Mooney–Rivlin and Yeoh models have been obtained by D Huri [8], showing that the three-term Yeoh model agreed better with the experimental results than that of the one-term model. Compared with the Neo-Hookean model, the Mooney–Rivlin model and the Ogden model were shown to be more applicable to chloroprene rubber in the specific ranges for the isotropic hyper-elastic model [9]. For rubberlike materials, a constitutive modeling was proposed by FA Denliv [10] with a data-driven hyper-elasticity approach; then, the stress–strain response could be constructed directly by using experimental data.

In 1972, Wright used a sinusoidal force to excite beams in free–free conditions and measured the displacement using a contactless optical probe. Damping is then measured by recording the oscillation decay [11].

Guild and Adams [12] believed that the clamping pressure of the coil on the beam might introduce cracks during the test of the instrument used by Adams and Bacon, thereby increasing the damping capacity and causing errors [13]. They propose two new apparatuses, one for free–free vibration and one for cantilever vibration. The coil clamp was improved to be stiffer to exclude spurious contributions to damping. The Specific Damping Capacity (SDC) was calculated using Adams and Bacon’s same method, and the trends of the two methods were found to be similar.

After verification, Talbot and Woodhouse found that laminate theory can accurately predict elastic behavior and generalized it to the prediction of damping characteristics, but the accuracy is only 30% [14].

The mechanism of modal damping at work in laminated FRP (fiber-reinforced polymer) composite panels was studied by Maheri. It was shown that the extent of the variation in modal damping over a range of modes and boundary conditions is generally a function of the extent of the variation of the laminate’s stiffness concerning direction [15]. In a follow-up study, the same effect was observed [16].

D.G. Fradkin first defined the damping function, D.F., to quantitatively analyze the relationship between molecular structure and damping capacity, which is defined as $D.F.=\int E^{\prime \prime }dT$. Later, M.C.O. Chang et al. used the area, LA, under the linear loss modulus E”-T curve and the area under the loss factor tanδ-T curve, TA, to describe the damping capacity of the material [17,18].

In this work, an experimental device fixed with a laser displacement sensor was assembled and the damping properties of ethylene-propylene-diene monomer (EPDM)/chlorinated butyl rubber (CIIR) blends were investigated. In addition, the effects of raw rubber ratios on the vulcanization characteristics, rebound behaviors and damping capacity were studied. Finally, a phenomenological method was proposed to characterize the damping capacity of EPDM/CIIR blends in accordance with the rebound behavior of rubber balls.

## 2. Experimental

### 2.1. Materials

Ethylene Propylene Diene Monomers (EPDM) used in this work was provided by Mitsui Chemical, Tokyo, Japan, and the ENB and ethylene contents were 4.1 wt% and 48 wt%, respectively. Chlorinated Butyl rubber (CIIR) with chlorine and isoprene contents of 1.25 wt% and 2.0 mol%, respectively, was purchased from Exxon Mobil Corporation, Irving, TX, USA. Carbon black (N550) with the particle size of ca. 50 nm was supplied by Cabot Co., Ltd, Boston, MA, USA. The curing system consisting of sulfur and 2,2′-dibenzothiazoledisulfde (DM), and activation system including stearic acid and zinc oxide were offered by Rhein Chemie Co., Ltd, Qingdao, China. Paraffin oil with the density of 0.899 g/cm^3^ (15 °C) and the relative molecular weight of 720, and N-1,3-dimethylbutyl-N′-phenyl-p-phenylenediamine (antioxidant 4020) were purchased from commercial sources.

### 2.2. Experimental Formula

The experimental formula is shown in Table 1.

### 2.3. Sample Preparation

According to the formula given in Table 1, CIIR and EPDM raw rubbers were blended firstly in an internal mixer for 1 min, then the zinc oxide, stearic acid and antioxidant were added and mixed further for 1 min. At last, the carbon black and paraffin oil were added and mixed for 4 min to obtain the masterbatch. The mixing temperature was set as 55 °C and the rotor speed was set to be 60 rpm. The obtained masterbatch was mixed with sulfur and DM by a two-roll mill with the roller speed ratio of 1:1.2 in order to obtain the compounds. After being kept at room temperature for 12 h, the compounds were vulcanized at 175 °C to obtain rubber ball samples with a diameter of 30 mm.

### 2.4. Characterization Methods

**Figure 1:**
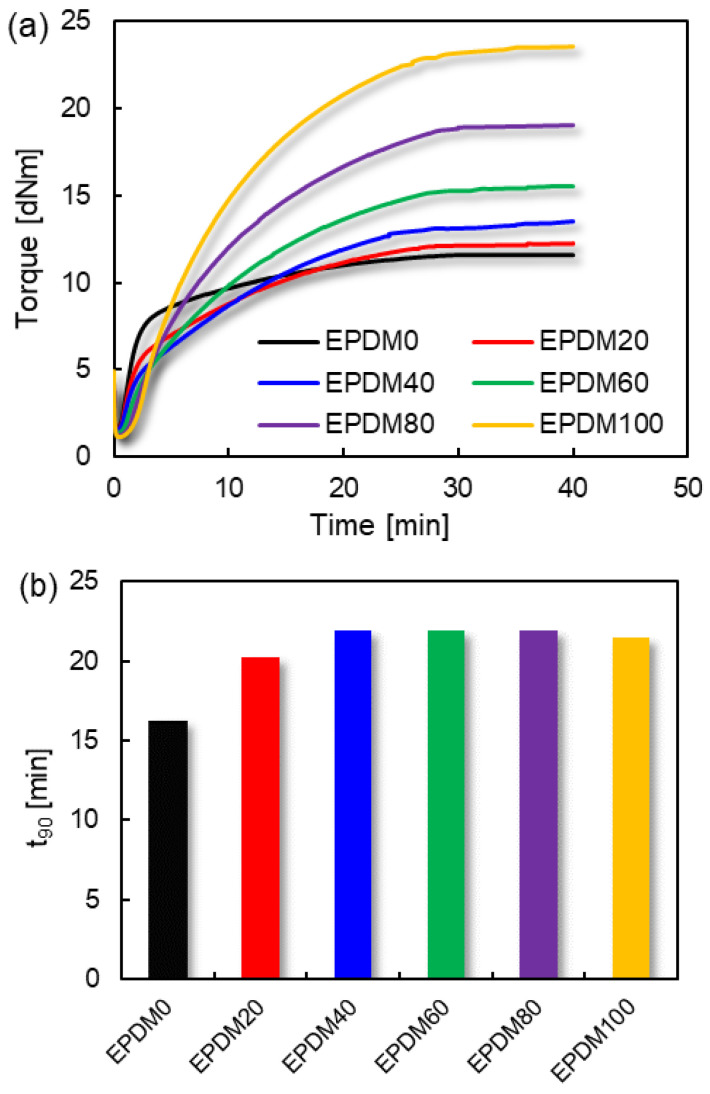
Vulcanization characteristics of EPDM/CIIR blends: (**a**) curing curves; (**b**) optimal curing time t_90_.

The curing characteristics of the CIIR/EPDM blends were performed with MDR test at 175 °C. Dynamic mechanical analyzer (DMA) was adopted to measure the relations of the loss factor (tanδ) with frequency. A laser displacement sensor, as shown in Figure 1, was used to collect the displacement information of the rubber ball samples for the purpose of determining the rebound behavior. The rubber ball samples were first placed 300 mm away from the ground. The laser displacement sensor samples 98 times per second, that is, the interval between the two points collected by the sensor is 1/98 s.

## 3. Results and Discussions

### 3.1. Curing Characteristics

The combination of more than two different rubbers is frequently used which can improve the deficiencies of existing rubber performance [19,20,21,22,23]. The vulcanization characteristics of EPDM/CIIR blends are shown in Figure 1.

As can be seen from Figure 1, CIIR has the fastest curing speed (minimum t_90_ value) but the lowest torque value. On the contrary, EPDM has the highest torque value but quite a slow curing speed. Simultaneously obtaining higher curing speed and curing torque is one of the main purposes of CIIR and EPDM blends, which can be beneficial to improve the performance of terminal products.

### 3.2. Damping Characteristics

#### 3.2.1. Damping Mechanism of Rubber Materials

The conformation and conformational entropy change significantly when the macromolecular rubber chain is subjected to force, and part of the energy input from the outside is stored. At the same time, the macromolecular chains rub against each other when they move under the action of external force, converting energy into heat and dissipating it to the outside world. The dissipation of this energy is called damping capacity. Therefore, when the energy input into the system from the outside is known, it is only necessary to measure the energy stored in the system and subtract the energy stored in the system from the energy input from the outside to obtain the energy lost by the system.

The gravitational potential energy of a rubber ball of mass *m* at height *h*_0_ is as follows:

(1) $$W_{G0}=mgh_{0}$$

where *m*—mass of the rubber ball;

*g*—gravitational acceleration;
*h*_0_—the height of the rubber ball when it is released;
*h_i_*—the *n*th rebound height of the rubber ball.

The gravitational potential energy transforms into kinetic energy when the rubber ball falls. The rubber ball will undergo macroscopic deformation due to the action of external force once the ball touches the ground. Microscopically, the macromolecule segments are forced to move, resulting in friction occurring between adjacent molecular chains. For the filled rubber system, there is friction contributed from the interaction of molecular chain and filler and of filler and filler [24]. Due to the internal friction of the rubber ball, it can only reach the height of *h*_1_ (*h*_1_ < *h*_0_) when it rebounds upward from the ground. At this time, the gravitational potential energy of the rubber ball becomes

(2) $$W_{G1}=mgh_{1}$$

Therefore, during the first rebound, the energy loss of the rubber ball is

(3) $$W_{loss}=W_{G0}-W_{G1}=mg(h_{0}-h_{1})$$

Therefore, the energy loss of a rubber ball in a rebound process is directly proportional to the difference between the initial and final height. The rubber ball will then repeat the above process until it stops due to energy depletion.

**Figure 2:**
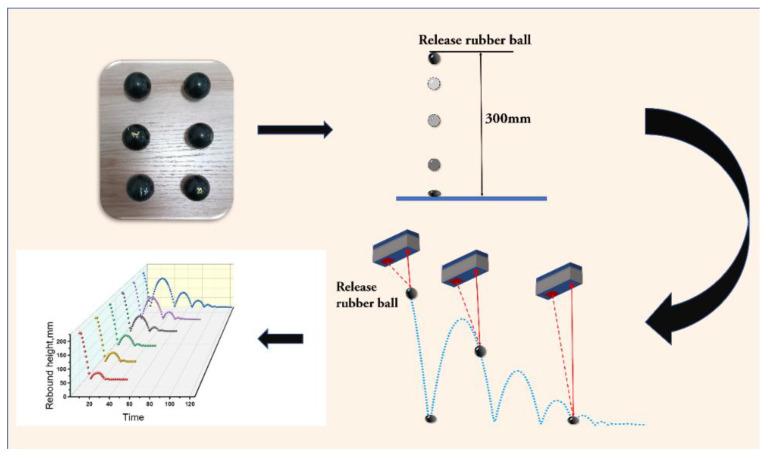
Schematic diagram of rubber ball rebound experiment process and experimental device.

Figure 2 shows the rebound experimental device of the rubber ball, which is composed of a laser sensor connecting to a computer. A rubber ball is placed under the sensor and the released rubber ball will move vertically up and down reciprocally. At the same time, the device captures the motion track of the rubber ball and displays it on the computer.

**Figure 3:**
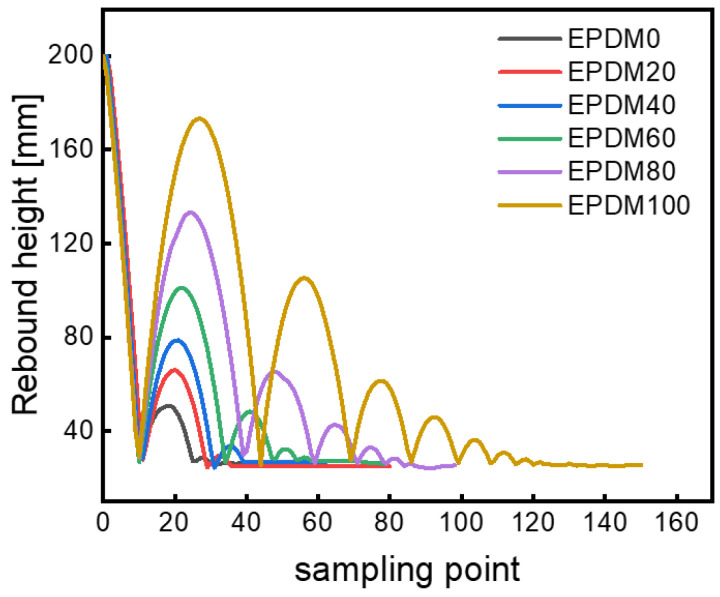
Results of the rebound behaviors of rubber balls.

The rebound behaviors of EPDM/CIIR blends are shown in Figure 3. The curve on the left of the wave peak represents the process of the rubber ball rebounding back from the ground to reach the highest point, and the curve on the right of the wave peak represents the process of the rubber ball falling from the highest point to reach the ground. Hence, the number of wave peaks indicates the rebound times of the rubber ball. In the end, the curve becomes a straight line, meaning that the rubber ball has been completely stationary, and the value of the vertical coordinate at this time indicates the diameter of the rubber ball. Two key points need to be emphasized here. Firstly, the release height of the ball is 300 mm, which has exceeded the range of the sensor, and Figure 3 only shows the data from 0 to 200 mm. Secondly, the horizontal coordinate of Figure 3 indicates the sampling point (which actually means the movement time of the ball), rather than the lateral movement of the ball during the rebound process. In summary, the rebound height, the number of rebounds, and the rebound time (the time experienced by the rubber ball from falling to a complete stop) of the rubber ball can be obtained from Figure 3.

#### 3.2.2. The Deformation Process of Rubber Ball

It is necessary to analyze the deformation process of the rubber ball in contact with the ground because the energy dissipation occurs in this process.

**Figure 4:**
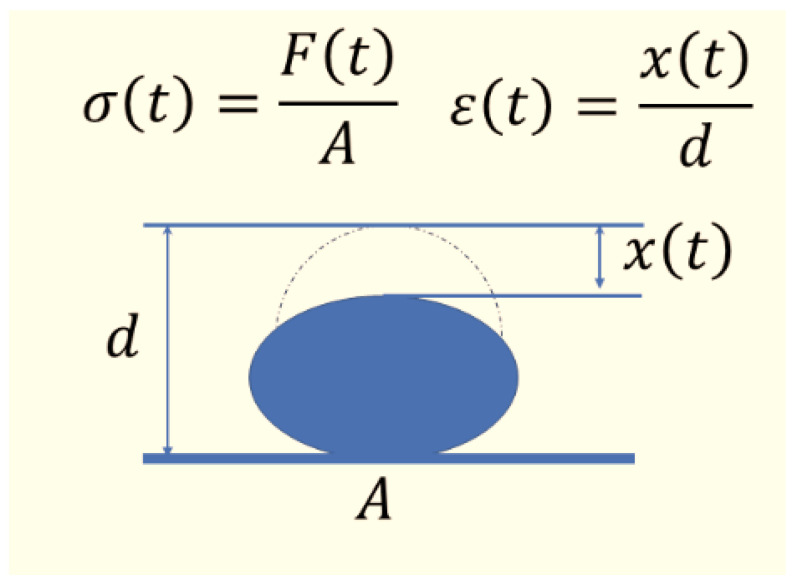
The deformation process of rubber ball. *σ*(*t*)— stress in the rubber ball during deformation. *ε*(*t*)— strain in the rubber ball during deformation. *F*(*t*)— the force on the rubber ball during the deformation. *A*—area of the rubber ball in contact with the ground during deformation. *x*(*t*)— travel distance of rubber ball vertices. *d*—diameter of the rubber ball.

Figure 4 shows the deformation process of the rubber ball. When the rubber ball touches the ground, it experiences negative acceleration *a*(*t*). The force acting on the rubber ball is *F*(*t*). Equation (4) can be obtained according to Newton’s formula

(4) $$F\left(t\right)=m\cdot a\left(t\right)$$

The force *F*(*t*) causes strain *ε*(*t*) and stress *σ*(*t*) of the rubber ball. When it is linearly deformed. The following Equation (5) is obtained where *A*(*t*) is the contact area between the ball and ground:

(5) $$F\left(t\right)=\sigma \left(t\right)\cdot A\left(t\right)=E\left(t\right)\cdot \varepsilon \left(t\right)\cdot A\left(t\right)=m\cdot a\left(t\right)$$

*E*(*t*)—Modulus of rubber balls.

The acceleration *a*(*t*) corresponds to the second-order derivative of the deformation distance, which has the following Equation (6):

(6) $$a\left(t\right)=\frac{dv\left(t\right)}{dt}=\frac{d^{2}x\left(t\right)}{d^{2}t}=x^{\prime \prime }\left(t\right)$$

Deformation of strain *ε*(*t*) is approximately obtained by the ratio of the deformation distance at the top of the rubber ball to the diameter of the ball, as depicted in the following Equation (7):

(7) $$\varepsilon \left(t\right)\approx \frac{x\left(t\right)}{d}\to \varepsilon^{\prime \prime }\left(t\right)\approx \frac{x^{\prime \prime }\left(t\right)}{d}$$

Equations (4)~(7) constitute a description of the deformation motion of the rubber ball when it contacts the ground.

(8) $$\frac{E\left(t\right)\cdot A\left(t\right)}{m\cdot d}\cdot \varepsilon \left(t\right)\approx \varepsilon^{\prime \prime }\left(t\right)$$

By substituting Equations (5) and (6) into Equation (7) one can obtain Equation (8), which can be solved when both *E*(*t*) and *A*(*t*) are known. *E*(*t*) varies with the type and proportion of the constituents in the rubber ball, and the magnitude of modulus *E*(*t*) effects the change in contact area *A*(*t*). The lower the modulus *E*(*t*), the larger the deformation of the rubber ball and thus the larger the contact area. The Equation (8) can be solved by the contact area *A*(*t*) and the modulus *E*(*t*), i.e., the deformation frequency of the rubber ball on contacting the ground. This means that the deformation frequencies of rubber balls differ for different materials even if the rubber balls are released at the same height. In summary, the rebound height of the rubber ball indicates the damping capacity of the rubber under a specific external excitation (the magnitude of the excitation is determined by the release height of the rubber ball), rather than the damping capacity at a specific frequency.

Additionally, the first rebound height of the pure EPDM ball is the highest, while the pure CIIR ball is the lowest (Figure 3). The rebound height decreases with the increase in CIIR content, indicating that the pure CIIR ball dissipates the most energy in the deformation process and has the greatest damping capacity due to the dense side methyl groups in the molecular chains of chlorobutyl rubber. The intense friction and strong energy dissipation between the CIIR molecular chains occur under external action. For the case of EPDM, however, the small number of methyl groups in the highly regular molecular chains results in less energy dissipation under external force, and thus the rebound height of the EPDM ball is the largest.

There are two ways to solve Equation (8). The first one is the finite element method, which is discussed in detail in the paper by R. Weiss. The second approach is to use constants to approximate the time-related parameters. Wrana analyzed the deformation process of a rubber ball in contact with the ground. The contact area *A*(*t*) and the modulus *E*(*t*) were hypothesized as constant values and the frequency of the periodic deformation of the rubber ball was further derived, as shown in Equation (9).

(9) $$\omega =\sqrt{\frac{E_{0}}{\rho d^{2}}}$$

*ω*—the frequency of the periodic deformation;
*E*_0_—Young’s modulus of rubber materials;
*ρ*—density of rubber balls;
*d*—diameter of rubber ball.

The frequency of the deformation of the rubber ball is related to *E*_0_, *ρ* and *d*. The rebound height of the rubber ball can be predicted by combining the master curve of the material at 20 °C and bringing the relevant parameters into Equation (9).

The method of approximating the variables as constants allowed the researchers to obtain the rebound height of the rubber ball only over a relatively wide range of frequency. In this paper, we propose a method to predict the rebound height of rubber balls from another perspective.

### 3.3. Prediction of the Rebound Height

According to the literature [25], when the rubber ball falls at the same height, the energy dissipated can be approximated as a loss factor tanδ with the following Equation (10).

(10) $$\frac{\tan\delta_{1}}{\tan\delta_{2}}=\frac{\Delta h_{1}}{\Delta h_{2}}$$

Then, Equation (11) is readily obtained by transforming Equation (10).

(11) $$\Delta h_{2}\cdot \tan\delta_{1}=\Delta h_{1}\cdot \tan\delta_{2}$$

Multiplying both sides of Equation (11) by *x* one can obtain Equation (12).

(12) $$x\cdot \Delta h_{2}\cdot \tan\delta_{1}=x\cdot \Delta h_{1}\cdot \tan\delta_{2}$$

Obviously, the following Equation (13) must be true.

(13) $$y\cdot \Delta h_{2}\cdot \tan\delta_{2}=y\cdot \Delta h_{2}\cdot \tan\delta_{2}$$

Hence, Equation (14) is obtained by adding Equation (12) to Equation (13).

(14) $$\left(x\cdot \tan\delta_{1}+y\cdot \tan\delta_{2}\right)\cdot \Delta h_{2}=\left(x\cdot \Delta h_{1}+y\cdot \Delta h_{2}\right)\cdot \tan\delta_{2}$$

Equation (15) is then obtained from simple transformation of Equation (14),

(15) $$\frac{\tan\delta_{2}}{x\cdot \tan\delta_{1}+y\cdot \tan\delta_{2}}=\frac{\Delta h_{2}}{x\cdot \Delta h_{1}+y\cdot \Delta h_{2}}$$

among them, making

(16) $$tan\delta_{3}=x\cdot \tan\delta_{1}+y\cdot \tan\delta_{2}\;\left(x+y=1\right)$$

*x*—mass fraction of rubber 1 in the raw rubber system of the blends.
*y*—mass fraction of rubber 2 in the raw rubber system of the blends.
tanδ_1_ and tanδ_2_—loss factors of rubber 1 and 2 at the same frequency, respectively.
tanδ_3_—loss factor of blends composed of rubber 1 and 2.
*Δh*_1_ and *Δh*_2_—the height differences before and after the rubber 1 and 2 ball rebound, respectively.

Equation (16) describes the loss factor of the blends as a function of the loss factor of the pure rubber.

**Figure 5:**
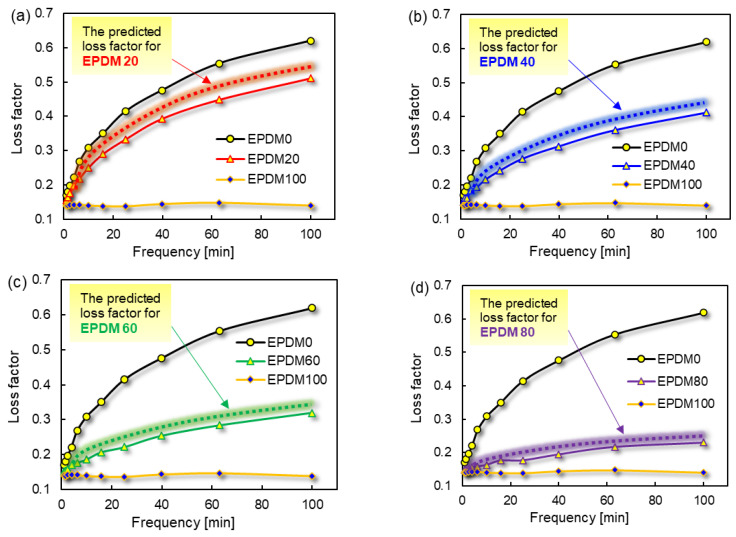
The measured and predicted tanδ of rubber blends. (**a**): The predicted loss factor for EPDM 20, (**b**): The predicted loss factor for EPDM 40, (**c**): The predicted loss factor for EPDM 60, (**d**): The predicted loss factor for EPDM 80.

The loss factors of pure rubber and blends were tested at frequency from 1 Hz to 100 Hz at room temperature. The loss factors of the rubber blends were predicted and compared according to Equation (16). The results are shown in Figure 5.

The loss factors of the rubber blends with EPDM content of 20 wt%, 40 wt%, 60 wt% and 80 wt% were predicted and correlated with frequency. Although the predicted value is slightly higher than the measured value, it is basically consistent with the experimental results. In the same way, *∆h* of the rubber ball should also follow the corresponding law. Thus, Equation (17) can be obtained.

(17) $$\Delta h_{3}=x\cdot \Delta h_{1}+y\cdot \Delta h_{2},\;\;\left(x+y=1\right)$$

By a simple conversion, it can be deduced that the rebound height of the rubber ball also conforms to Equation (17), and thus Equation (18) is obtained.

(18) $$h_{i}=h_{E}x+h_{C}y,\;\;\left(x+y=1\right)$$

*h_i_*—first rebound height of the rubber ball with EPDM content i in the raw rubber system.
*h_E_*—the first rebound height of pure EPDM ball.
*h_C_*—the first rebound height of pure EPDM ball.
*x*—mass fraction of EPDM in raw rubber *i*.
*y*—mass fraction of CIIR in the raw rubber (1 − *i*).

The rebound heights of the theoretical (*hi*) and measured (*Hi*) values and the difference between *Hi* and *hi* for the rubber balls are listed in Table 2.

**Figure 6:**
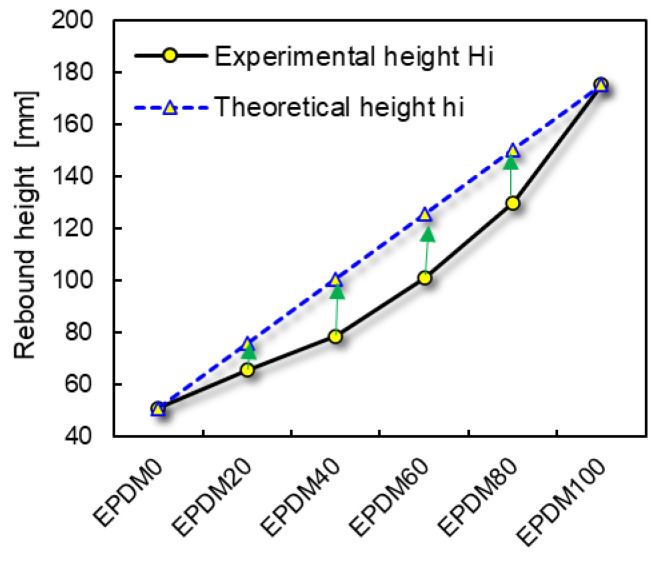
Theoretical and measured rebound heights of the rubber ball.

Figure 6 shows the difference of the theoretical and measured rebound heights (*hi* − *Hi*) firstly increases and then decreases with the increase in EPDM content in the raw rubber system. In other words, the larger the difference between the two rubber ratios, the lower the error of the Equation (18).

The deviation of the theoretical rebound height can be explained by the following reasons. The migration of the vulcanizing agent due to the different solubility in EPDM and CIIR leads to a change in the crosslinking density of the two rubber phases in blends compared with that of the pure rubber [26]. The rubber phase with high solubility to the vulcanizing agent moves to a higher glass transition temperature (Tg) because the crosslinking density becomes larger, the chain segment movement becomes more impeded and the free volume is thus further reduced. The Tg of the other rubber phase moves to lower temperatures, hence the damping capacity of the rubber blend changes.

Thus, Equation (18) presupposes that the crosslinking density of the two rubber phases in the rubber ball with EPDM content *i* is the same as that of the respective single rubber. In this work, the different solubility of the vulcanizing agent in the two rubber leads to changes in the crosslinking density of the two rubber phases in the blends, which causes changes in the damping properties [27]. This is the reason for the deviation of the measured rebound height from the theoretical calculation. Therefore, a modified Equation (19) is obtained by adding a correction term *c* into Equation (18) as following:

(19) $$h_{i}=h_{E}x+h_{C}y+c$$

*c* denotes the difference between the theoretical dynamic mechanical properties and the actual dynamic mechanical properties of the material with EPDM content *i*. In this experiment, the predicted loss factor is smaller than the actual one.

As discussed above, we have obtained the knowledge that the rebound height represents the damping capacity of the rubber ball under specific excitation (the value of the excitation is determined by the release height of the rubber ball). In other words, each ball receives the same excitation when it falls at the same height. The deformation frequencies, however, are varied with the rubber balls of different blending ratios. In addition, it is difficult to determine the value of the correction term *c* based on the frequency-dependent measurement data at room temperature because the deformation frequency of the ball is hard to determine.

In order to determine the value of *c*, the method of taking the average value is adopted in this paper.

Assume that

(20) $$c=-1/2\times \left(24.4+21.9+20+20.1\right)=-21.6,$$

then, Equation (21) is obtained.

(21) $$h_{i}=h_{E}x_{E}+h_{C}x_{C}-21.6$$

Actually, the correction term *c* is determined by various factors; for example, the nature of the rubber and formulations. Thus, it reveals various values for different kinds of rubber blends which will be discussed in detail in the follow-up work. Equation (21) provides a simple method to predict the damping capacity of a rubber that is measured by the rebound height and plays a key role in the formulation design of viscoelastic damping rubber materials.

### 3.4. Rebound Time of the Rubber Ball

**Figure 7:**
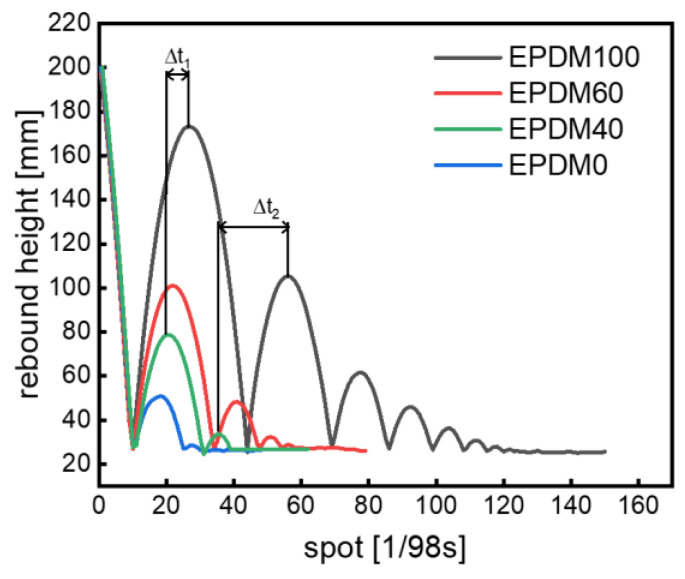
The rebound time interval of rubber balls.

The maximum heights of the rubber balls rebounding to the highest point at the first time are different for various rubber blends, and hence the deformation frequency and external excitation of the ball falling to the ground again are different, subsequently resulting in a series of different rebound heights (Figure 7). The peaks indicate the highest rebound points of the rubber balls. The difference between the horizontal coordinates of the *n*th peaks of different balls represent the difference of the time elapsed (∆t) between the two balls when they reach the highest point for the *n*th time.

As shown in Figure 7 again, the distance between the two peaks increases with the increase in the rebound times of the rubber ball. This means that in rubber balls made of different rubber mixtures, even if the number of rebounds is the same, the rebound time is different. Therefore, the rebound time of the rubber balls is also an important parameter to characterize the damping capacity of materials. The shorter the rebound time, the faster the rubber ball dissipates energy, that is, the better the damping capacity.

**Figure 8:**
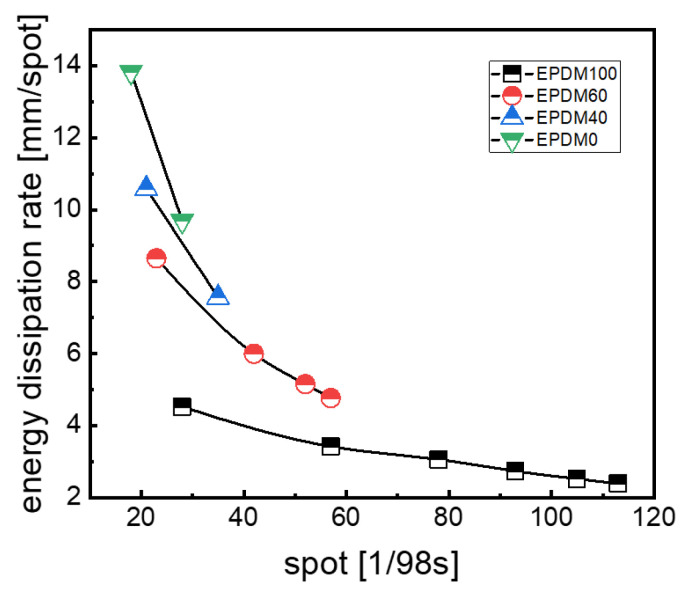
EDR of rubber balls.

The difference between the initial height of the rubber ball and the *i*th rebound height (*h*_0_ − *h_i_*) is defined as the height loss. What is noteworthy is that the height loss and the rebound time take no account of the specific bouncing process. The Energy Dissipation Rate (EDR) of the rubber ball can thus be defined and obtained by dividing the height loss by the rebound time (Figure 8).

The changes in the EDR with time for the rubber balls based on different rubber blends are described in Figure 8. By longitudinally comparing the EDR values of each sample with time we can find that both the EDR of the rubber ball and the time interval between each two data points decrease gradually. It can be concluded that the damping capacity decreases with the decrease in external excitation of the rubber ball when it falls within 300 mm, and the time interval between two adjacent rubber balls reaching the highest point becomes smaller due to the gradual decrease in the rebound height during the bouncing process. It also indicates, as shown in Figure 8, that the EDR value is the highest for the pure CIIR and lowest for the pure EPDM, showing a decreasing trend with the increase in EPDM content in the rubber blends.

## 4. Conclusions

The damping mechanism, rebound behavior, and deformation process of the rubber ball in contact with the ground were analyzed in detail. The damping capacity of the EPDM/CIIR rubber blends could be represented by the rebound height under a specific external excitation, the magnitude of which was determined by the release height of the rubber ball rather than the specific frequency. Under the experimental conditions, the determined damping capacity showed the highest value for the pure CIIR and the lowest value for the pure EPDM, revealing better damping performance with more CIIR content in the rubber blends. The prediction model was proposed and modified in the form of the equation to predict the rebound height of the rubber blends-based balls. The correction term in the equation represented the difference between the theoretical and the actual dynamic mechanical properties, which was caused by the difference in solubility of the vulcanization system in the two rubber phases. The introduction of the energy dissipation rate (EDR), defined by combining the height loss with the rebound time, could be employed to characterize the damping capacity of the rubber blends. The rubber products with more CIIR content would cause more energy loss and exhibit a more effective damping capacity.

## Figures and Tables

**Table 1:** The details of compound formulas and formula codes.

Ingredient (phr)	EPDM0	EPDM 20	EPDM 40	EPDM 60	EPDM 80	EPDM 100
CIIR	100	80	60	40	20	
EPDM		20	40	60	80	100

Other ingredients (phr): Carbon black (N550) 50, Paraffin oil 5, Zinc oxide 5, Stearic acid 2, Antioxidant (4020) 1, Sulfur 2, DM 1.

**Table 2:** The theoretical (*hi*) and measured (*Hi*) rebound heights of the rubber balls.

Rebound Height	EPDM	EPDM80	EPDM60	EPDM40	EPDM20	CIIR
*H_i_*/mm	175.3	130	101.1	78.7	65	50.9
*h_i_*/mm	175.3	150.4	125.5	100.6	75.6	50.9
(*hi* − *Hi*)/mm	0	20.4	24.4	21.9	10.6	0

## Data Availability

The original contributions presented in the study are included in the article, further inquiries can be directed to the corresponding authors.
